# Yunvjian Improves Glucose and Insulin Function in Diabetic Rats by Regulating Gastric Emptying Function

**DOI:** 10.1155/2023/8551406

**Published:** 2023-01-14

**Authors:** Wan-Yu Luo, Lin Gao, Dan-Dan Zhao, Lin Zhang, Bo Gao, Guang Lei, Guang-Tong Dong, Jun-Ping Wei

**Affiliations:** ^1^Department of Traditional Chinese Medicine, Beijing University of Chinese Medicine, Beijing, China; ^2^Department of Traditional Chinese Medicine, Shandong Traditional Chinese Medicine University, Shandong, Jinan, China; ^3^Department of Nuclear Medicine, Guang'anmen Hospital of China Academy of Chinese Medical Sciences, Beijing, China; ^4^Department of Endocrinology, Guang' anmen Hospital of China Academy of Chinese Medical Sciences, Beijing, China

## Abstract

**Background:**

Diet acts on the human body through digestion in the stomach and absorption in the intestines. Thus, the emptying of the stomach should be the focus of the research mechanism of the combined medicine and food treatment of diabetes. The emptying function of the stomach and the secretion of related hormones may be the key points of traditional Chinese medicine. In the clinic, Yunvjian is a famous traditional Chinese formula for preventing and curing diabetes. However, the pharmacological action and mechanism of Yunvjian are also need to be probe.

**Objective:**

To assess the effect of Yunvjian on glucose, insulin level and gastric emptying function and related hormones on high-fat diet combined with STZ-induced diabetic rats.

**Methods:**

High-fat diet combined with STZ was used to construct type 2 diabetes mellitus (T2DM) rats model and received a 4-week Yunvjian administration. The animals were divided into 6 groups, respectively, as the Control group, the DM group, the DM + Acarbose group, the DM + YNH group, and the DM + YNL group. Radionuclide single-photon emission computed tomography (SPECT) technology was used to observe the gastric emptying rate and half-empty time; blood was took to test fasting insulin, and then the insulin resistance index (HOMA-IR) was calculated; HE staining was performed to detect islets and gastric antrum, immunohistochemical staining was performed to detect the number and morphology of pancreatic *β* cells and gastric antrum Cajal cells, and the average optical density was calculated; the expression of ghrelin hormone in gastric antrum and serum was detected by ELISA and immunofluorescence; the expression of GHRS mRNA in gastric antrum was detected by RT-PCR method.

**Results:**

Yunvjian could significantly improve the glucose level and insulin function of rats. Compared with the DM group, Yunvjian was beneficial to low fasting blood glucose (FBG) (*P* < 0.01), increased glucose tolerance, and improved islet function at the same time (*P* < 0.05). At the same time, compared with the DM group (25.02 ± 0.05, 44 ± 12.33), the emptying rate of the DM + YNH group was significantly faster (64.98 ± 0.12), and the half row time was shortened (26 ± 8.29, *P* < 0.05). The gastric ghrelin levels in each group of Yunvjian increased with different degrees compared with the DM group (616.2 ± 26.23), especially in the DM + YNH group (863.51 ± 23.76, *P* < 0.01). Correspondingly, the expression of gastric GHSR mRNA in the DM + YNH and DM + YNL groups increased significantly compared with the DM group (*P* < 0.01).

**Conclusions:**

Yunvjian can effectively control glucose and improve islet function, which may be closely related to its influence on gastric emptying function and related hormone secretion regulation.

## 1. Introduction

The incidence of type 2 diabetes mellitus (T2DM) worldwide and the number of patients is increasing year by year, which leading the cause of death and disability worldwide [1]. The research report in 2019 showed that there were 463 million and 374 million patients with diabetes and impaired glucose tolerance between 20 and 79 years old, and the urban-rural distribution and age distribution of DM patients were not optimistic [2]. In China, nearly 10% of the urban population over the age of 20 are diabetic patients, and 70% of them have unsatisfactory glucose control [3]. Therefore, it is an important measure to improve T2DM to prevent and treat diabetes through multiple channels instead of relying solely on traditional hypoglycemic drugs.

Traditional Chinese medicine (TCM) has many advantages in lowering blood glucose and regulating lipid metabolic disorders in the treatment of DM [4, 5]. Herbal medicine can provide a simpler and more natural way to control DM without any side effects. Some herbs have shown useful antidiabetic effect and related mechanisms of action [6], such as American Ginseng and Astragali Radix, they are widely used in treatment of DM, and the mechanism is deeply studied [7, 8].

At present, various studies have been carried out to determine the pathogenesis of DM [9, 10]. Gastrointestinal motility disorder is a common complication of diabetes, accounting for about 40% of diabetic patients [11]. Gastrointestinal discomfort not only affects the patient's compliance with oral hypoglycemic drugs and aggravates the condition of diabetes [12]. The literature reports on diabetic gastrointestinal function are mostly in the later stage of diabetic gastroparesis [13], and the research reports on how to regulate blood glucose through gastric emptying function are limitedly.

Yunvjian is a classic TCM prescription, which contains five medicinal materials: *Rehmannia glutinosa* Libosch (Shu Di Huang), *Achyranthes bidentata* Bl (Niu Xi), Gypsum Fibrosum (Shi Gao), *Anemarrhena asphodeloides* Bge (Zhi Mu), and *Ophiopogon japonicus* Ker-Gawl (Mai Dong). Although there are more and more evidences from clinical and epidemiological studies [14, 15] showing that Yunvjian can effectively improve glucose in diabetic patients, the mechanism are still needed to explore. Previous researches have proved that the main medicinal ingredients in Yunvjian have the effects of lowering blood glucose [16] and improving gastrointestinal function [17]. The motive for conducting this study is to evaluate the effect of Yunvjian on diabetic rats induced by high-die fat (HDF) combined with streptozotocin (STZ), and to further explore the improvement of gastric emptying function of Yunvjian.

## 2. Materials and Methods

### 2.1. Animals

Four-week-old male SD rats, weighing about 100–150 g, were purchased from Beijing HFK Bioscience Co., Ltd. and raised in the SPF laboratory of Guang'anmen Hospital, China Academy of Chinese Medical Sciences (license number: SYXK (Jing) 2014-0041, the license of the Animal Ethics Committee: BUCM-4-2017101201-4014). Adaptation feeding for 3 days before the formal experiment. The ambient temperature is controlled at 20–25°C, the humidity is 55% ± 5%, normal light (lighting time: 7:00–19:00), free to take food and drinking water. All experimental operations procedures and feeding conditions are in accord with the “Guidelines for the Management and Use of Laboratory Animals,” and each experimental link has tried its best to reduce the suffering of experimental animals.

### 2.2. Drugs

Yunvjian contains five medicinal materials: *Rehmannia glutinosa* Libosch (Shu Di Huang), *Achyranthes bidentata* Bl (Niu Xi), Gypsum Fibrosum (Shi Gao), *Anemarrhena asphodeloides* Bge (Zhi Mu) and *Ophiopogon japonicus* Ker-Gawl (Mai Dong). Provided by the Department of Pharmacy, Guang'anmen Hospital, China Academy of Chinese Medical Sciences. After soaking in cold water for half an hour, decocted, concentrated into 2 g/mL liquid medicine, filtered, and sterilized with 0.22 *μ*m filter. We calculate the equivalent dose for rats based on the conversion factor of human-rat body surface area of 6. Dosage of Yunvjian for adults is 60 g, equivalent to 6 g/kg of rat crude drug. Therefore, rats need 24 g/kg of rat crude drug daily.

### 2.3. Experimental Materials

Streptozotocin (STZ) purchased from Sigma, Germany. Eosin dye was purchased from Wuhan Saiweier Biological Technology Co., Ltd. Hematoxylin dye was purchased from Beijing Solarbio Technology Co., Ltd. Insulin Receptor *β* (E9L5V) XP® Rabbit mAb was purchased from CST, USA. HRP-labeled goat antirabbit IgG was purchased from ZSGB-BIO, Beijing. Insulin detection kit (radioimmunoassay) was purchased from Beijing Northern Institute of Biology Co., Ltd. 99 mm TC-DTPA was purchased from Beijing Atom High-Tech Company, with radiochemical purity ≥95%. Ghrelin kit was purchased from Shanghai Enzyme Link Biotechnology Co., Ltd. Anti-c-kit antibody [EPR22566-344] purchased from Abcam, UA.2 × SYBR Green qPCR Master Mix (Low ROX) purchased from Service bio Technology Co., Ltd., Wuhan.

### 2.4. Experiment Grouping and Design

Exclude rats with glucose ≥11.1 mmol/L. Randomly assign 10 rats to the control group for regular feeding, the others were fed with a high-fat diet (fat intake was 60% kcal), and the high-fat diet was produced by Beijing HFK Bioscience Co., Ltd. for 4 weeks. After 4 weeks, the rats were fasted overnight (without water) for 12 h, and each animal was given 3 intraperitoneal injections of 1% streptozotocin at a dose of 20 mg/kg, 15 mg/kg, and 15 mg/kg, with an interval of 4 h between each injection. After 3 days, all rats were tested for FBG, and FBG ≥11.2 mmol/L was determined as a T2DM model, and they were included as experimental animals in this experiment. Rats that did not meet the standard were supplemented with 10 mg/kg 1% STZ or eliminated. We continue feeding with high-fat feed for about a week to stabilize glucose. We select 40 T2DM rats and randomly divide them into the following 4 groups, each with 10 rats: DM group, DM + YNH group (Yunv 24 g/kg), DM + YUNVL group (YUNV 12 g/kg), and DM + Acabose (Acabose 10 mg/kg). The rats in each group are treated by gavage every day for 4 weeks. The control group and DM group are given the same amount of distilled water. During the experiment, the FBG was measured at the same time before the administration, the 1st, the 2nd, the 3rd, and the 4th week after the drug. Acupuncture points is selected from the tip of the tail, and the average blood glucose value of each rat is tested twice. After the blood collection, erythromycin ointment was applied to the wound site to prevent infection. We change the litter before each test to prevent the rat from hiding food in the litter from affecting the blood sugar level. The rat cannot help water for 12 h without food. After the last administration, rats in each group are fasted for 12 h, and the oral glucose tolerance test (OGTT) is performed. 1% pentobarbital sodium 3 mL/kg was injected intraperitoneally for anesthesia, the whole abdomen was opened after fixation, blood was taken from the abdominal aorta, and 2 mL was collected from the coagulation tube, left standing at room temperature for 30 min, and centrifuged at 2,500 r/min for 15 min, then take the upper serum. Extract rat islets and gastric antrum tissues. After washing with PBS, the islets and a part of the gastric antrum tissue are immersed in formalin and stored at room temperature; the other part of the gastric antrum tissue is divided into cryopreservation tubes and stored in a refrigerator at −80°C for later use. Serum is used for further biochemical analysis of insulin, and the homeostasis model assessment-insulin resistance (HOMA-IR): HOMA-IR = fasting serum insulin (mU/mL) × fasting blood glucose (mmol/L)/22.5. This research were conducted according to the National Center Animal Experimentation Committee on the care and use of laboratory animals. The experimental scheme was approved by the Animal Ethics Committee of Guang'anmen Hospital of China Academy of Chinese Medical Sciences.

### 2.5. Oral Glucose Tolerance Test

Four weeks after administration, rats were fasted for one night, and test the oral glucose load at 2 g/kg body weight. Blood glucose level was measured before and 15, 30, 60, 90, and 120 min after glucose administration via the tail vein. The areas under the curve (AUCs) were calculated and used to assess glucose tolerance.

### 2.6. Radionuclide SPECT Technology to Detect Gastric Emptying Residual Rate and Emptying Rate

At 4 weeks, the rats were deprived of food for 12 h and water for 6 h. Animals were fixed on the rat board without anesthesia lying on their back. After fixation, we take 4 mL of pure milk labeled with nuclide technetium (99 m Tc-DTPA 0.5 m Ci). After gavage, the gold standard for measuring gastric emptying-radionuclide SPECT measurement is used. We use 4 mm pinhole collimator front single-photon emission computed tomography to perform static planar imaging of the abdomen every minute. The center of the pinhole is vertically aligned with the abdomen of the rat and the distance is 5 cm. The matrix is 128 × 128, and the acquisition magnification is 3.2, one frame is collected every minute for a total of 60 frames. After the image acquisition is completed, the time-radiation curve is drawn as the gastric emptying curve, and the gastric emptying special software is used for data processing to obtain the total gastric half-emptying time (GET1/2). Gastric emptying rate (%) = total radioactive element content − minimum radioactive element content within 60 min/total radioactive element content.

### 2.7. Histopathological Changes of Islets and Gastric Antrum

After washing the tissue with running water, we place the tissue in the embedding frame, dehydrate it from low-concentration alcohol to high-concentration alcohol, and put it in xylene for transparency. We pour the melted paraffin into the embedding frame and use heated tweezers to put the tissue block in. We slice 8 *μ*m and bake slices at 60°C for 15 min. Xylene deparaffinization (10 min/time, 2 times), washed with PBS after hydration with gradient alcohol. 50 *μ*L of 1*X* hematoxylin and eosin staining solution for HE staining, and sealing with neutral gum. We place the islets and gastric antrum tissue morphology under an optical microscope, and collect images for analysis.

### 2.8. Immunohistochemical Observation of the Expression and Morphology of Cajal Cells and Insulin *β* Cells

We take the tail of islets and gastric antrum 0.5 × 0.5 cm, follow the above method to paraffin slices dehydrated, microwave oven antigen heat repair, put the slices in 3% hydrogen peroxide solution, and incubate at room temperature for 25 min in the dark. We add 3% BSA dropwise to cover the tissue uniformly in the histochemical circle, and seal at room temperature for 30 min. We add anti-c-kit and Insulin Receptor *β* (E9L5V) XP® Rabbit mAb in a ratio of 1 : 100 with PBS dropwise, and lay the slices flat in a humidified box and incubate them overnight at 4°C. The next day, 1 : 2500 diluted IgG was dropwise added to cover the tissue and incubated it at room temperature for 50 min. We add freshly prepared DAB chromogenic solution to the circle, dehydrate, and mount the slides, and then we examine it under a microscope, and then image acquisition and analysis. The nucleus stained with hematoxylin is blue, and the positive expression of DAB is brown-yellow.

### 2.9. Detection of Ghrelin Content in Gastric Antrum Tissue by Immunofluorescence Method

The tissue is embedded in the quick-freezing table of the cryostat, the thickness of the section is 8–10 *μ*m, and the anti-Ghrelin antibody prepared with PBS in a ratio of 1 : 100 is dropped on the slice, and incubated at 4°C overnight. After washing 3 times with PBS, we add DAPI dye solution to the circle, wash 3 times with PBS, mount the antifluorescence quenching mounting plate, and incubate at room temperature in the dark for 10 min. We observe and collect images under a fluorescence microscope.

### 2.10. Biochemical Assay

According to the instructions, we use insulin (Ins) radioimmunoassay kit and Ghrelin ELISA kit to detect serum insulin and Ghrelin levels, respectively.

### 2.11. RT-PCR Determination of Gastric GHSR mRNA Level

RNA was extracted and its absorbance was measured by spectrophotometer (NanoDrop2000). The concentration of RNA was measured by absorbance method. DNA was synthesized and quantified by using reverse transcription. The primer is shown in Table 1. PCR method is as follows: 3 min enzyme activation step at 95°C, 20 cycles, 15 s at 98°C, 30 s at 50°C, 40 s at 72°C, and 10 min at 72°C.

### 2.12. Statistical Method

Software SPSS 21.0 was used to process data. The results are expressed as “mean ± SE.” Paired or two groups of continuous measurement data were analyzed by *t*-test, one-way ANOVA was used for comparison among groups, and 4 evidence-based complementary, *P* < 0.05 means the difference is statistically significant, and *P* < 0.01 representing significant difference.

## 3. Results

### 3.1. Effect of Yunvjian on FBG, OGTT, and Insulin

**Figure 1:**
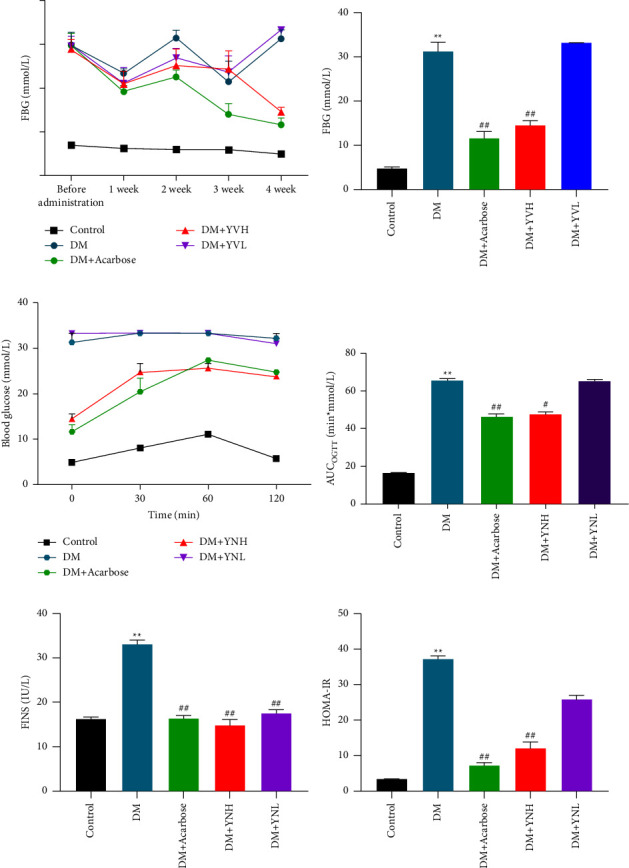
The effect of Yunvjian on FBG, OGTT, and IR: (a) 4-week trend of FBG level; (b) 4th week FBG level; (c) OGTT; (d) AUC_OGTT_; (e) FINS; (f) HOMA-IR. Data are expressed as mean ± standard error. Compared with the control group: ^*∗∗*^*P* < 0.01; compared with the DM group: ^#^*P* < 0.05 and ^##^*P* < 0.01.

FBG was measured every week. After the successful modeling, the blood glucose levels of the DM group, DM + acarbose group, DM + YNH, and DM + YNL groups were significantly higher than those of the control group (*P* < 0.01) (Figures 1(a) and 1(b)). In the first week of intragastric administration, compared with DM group, each administration group began to show the trend of lowering glucose. In the 4th week, compared with DM group, the FBG of the DM + YNH group was significantly lower than that of the DM group (*P* < 0.01), while the blood glucose level of the DM + YNL group was not statistically different from that of the DM group (*P* > 0.05).

As the results are presented in Figure 1(c), compared with the control group, the blood glucose level of the DM group was significantly higher before and after gavage (*P* < 0.05). At 30 min, the blood glucose of each group began to rise, while the DM group get peak after a meal and did not drop continuously. At 60 and 90 min, the DM + YNH group was significantly different from the DM group. Accompanied by significant decrease of AUC levels (*P* < 0.05, Figure 1(d)). Similarly, at 30, 60, 90, and 120 min, significantly lower glucose levels were observed in the DM + YNH group (*P* < 0.05).

Figures 1(e) and 1(f) show that, compared with the DM group, all treatment groups are significantly lower (*P* < 0.01). In contrast, the HOMA-IR value of the DM + YNH group was significantly reduced compared with the DM group (*P* < 0.05); the DM + YNL Yunvjian group showed a decreasing trend but no statistical difference (*P* > 0.05). Suggested that Yunvjian can reduce glucose tolerance, reduce insulin resistance, and retard the occurrence of hyperglycemia.

### 3.2. Effect of Yunvjian on Islets and Insulin *β* Cells

**Figure 2:**
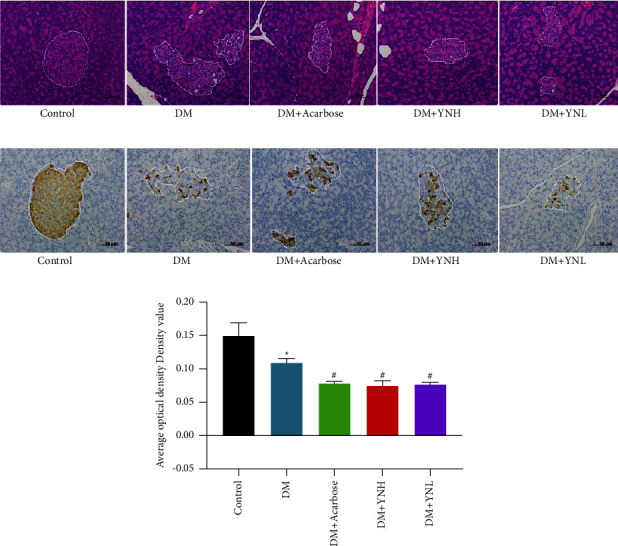
The effect of Yunvjian on the islets and pancreatic islets *β*-cell: (a) HE staining of islets (200×); (b) pancreatic islets *β*-cell immunohistochemical (200×); (c) pancreatic islets *β*-cell average optical density. The data are expressed as mean ± standard error of mean. Compared with the control group: ^*∗*^*P* < 0.05; compared with the DM group: ^#^*P* < 0.05.

HE showed that the number of islets in the DM group, the number of islets is reduced, the area is atrophied, the arrangement is irregular, the boundary with the islets is unclear, the structure is disordered, the cytoplasm in the islets is less, the exocrine glands invade inward, and part of the nucleus shrinks and divides. In the DM + YNH group is similar to DM + Acarbose, the pancreatic islets are slightly atrophy, the shape is approximately regular, and the boundary with exocrine is relatively clear (Figure 2(a)).

Immunohistochemistry showed that the number of pancreatic islet *β* cells in the DM group was significantly compensatory increased, the islet *β*-cells were sparsely arranged and unevenly distributed, the average optical density expression was significantly lower than that of the control group (*P* < 0.05). The number of *β* cells in the DM + YNH group was higher than that of the DM + Acarbose group, but there was no statistical difference in the average optical density value (*P* > 0.05, Figures 2(b) and 2(c)). There was no significant change in the DM + YNL group compared with the DM group (*P* > 0.05).

### 3.3. Effect of Yunvjian on Gastric Antrum and Cajal Cells

**Figure 3:**
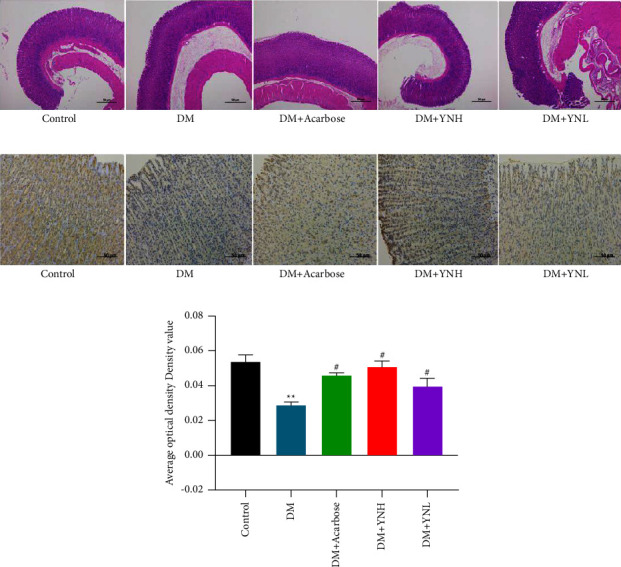
The effect of Yunvjian on gastric antrum and cajal cell: (a) HE staining of gastric antrum (40×); (b) c-kit immunohistochemical (200×); (c) c-kit average optical density. The data are expressed as mean ± standard error of mean. Compared with the control group: and ^*∗∗*^*P* < 0.01; compared with the DM group: ^#^*P* < 0.05.

Figure 3(a) shows the gastric mucosa, submucosa, muscularis, and serous membrane of the gastric antrum of rats have clear structures in the control group, and mucosal cells are arranged regularly and tightly, and no pathological changes. In the DM group, the arrangement of cells in the mucosal layer of gastric antrum was loose and chaotic, the intercellular space was enlarged, and the muscular ring muscle was obviously thinned. The DM + YNH group was similar to DM + Acarbose, with reduced intercellular space, no vacuole formation, and no telangiectasia.

C-kit receptor is currently the internationally used immunohistochemical staining method to mark the target protein of Cajal cells. The morphology was spindle-shaped with brown-yellow marks. The number of c-kit in the DM group significantly decreased than the control group (Figure 3(c), *P* < 0.05), the cells were atrophy, and the staining was light yellow. In the DM + Acarbose group and the DM + YNH group, the number of cells decreased slightly, but the morphology was normal. The DM + YNL group gradually became paler, less in number, and abnormal cell morphology (Figure 3(b)).

### 3.4. The Effect of Yunvjian on Gastric Emptying Function

**Figure 4:**
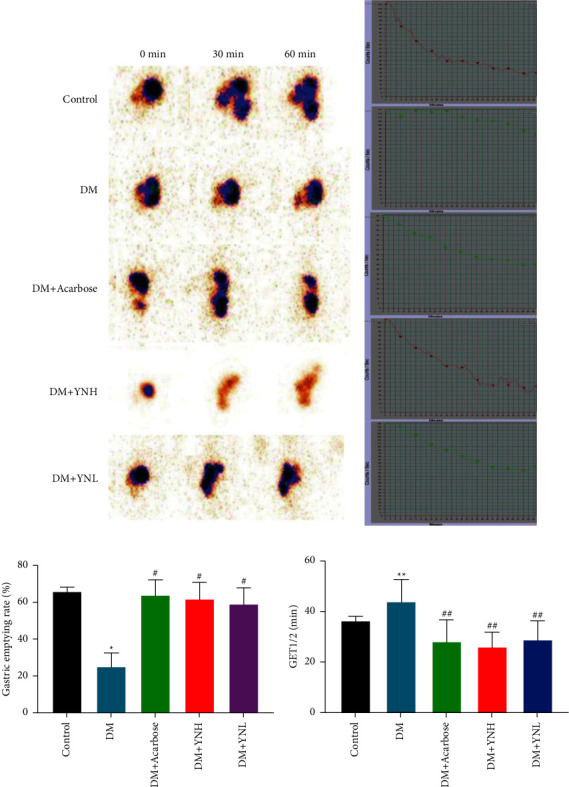
The effect of Yunvjian on gastric emptying function. (a) Gastric emptying calorie chart; (b) gastric emptying-time curve chart; (c) 60 min emptying rate; (d) half-emptying rate. The data are expressed as mean ± standard error of mean. Compared with the control group: ^*∗*^*P* < 0.05 and ^*∗∗*^*P* < 0.01; compared with the DM group: ^#^*P* < 0.05 and ^##^*P* < 0.01.

From Figure 4(a), it can be seen that the emptying rate of each administration group was significantly faster than that of the DM group after administration. Compared with the control group, the emptying rate of the DM + YNH group was basically close to or even faster. The half-draining time is similar to the emptying rate. Compared with the control group, the half-draining time of the DM group is significantly slower (*P* < 0.01). Compared with the DM group, the half-draining time of DM + Acarbose group and DM + YNH group is significantly faster (*P* < 0.05), the DM + YNL group also had a more obvious tendency to accelerate emptying, but there was no significant difference compared with the DM group (*P* > 0.05, Figure 4(c)).

The gastric emptying-time curve of the six group is showed in Figure 4(d). The horizontal axis in the figure is time, and the vertical sleeve is the radioactive element count in the stomach. In the figure, the gastric half-emptying time of rats in the control group is 36 min, and the gastric emptying rate of radionuclides is 55.73%; the emptying curve of rats in the DM group is disordered, and the half-exhaustion is not reached, and the gastric emptying rate of radionuclides is 25.81%; The gastric half-emptying time of rats in the DM + Acarbose group was 13.5 min, and the gastric emptying rate of nuclide was 75%. In the DM + YNH group, the gastric half-emptying time was 23 min, and the nuclide gastric emptying rate was 79.09%; the DM + YNL group was 42.5 min, and the nuclide gastric emptying rate was 51.79%.

### 3.5. Yunvjian on Ghrelin Hormone and GHSR mRNA

**Figure 5:**
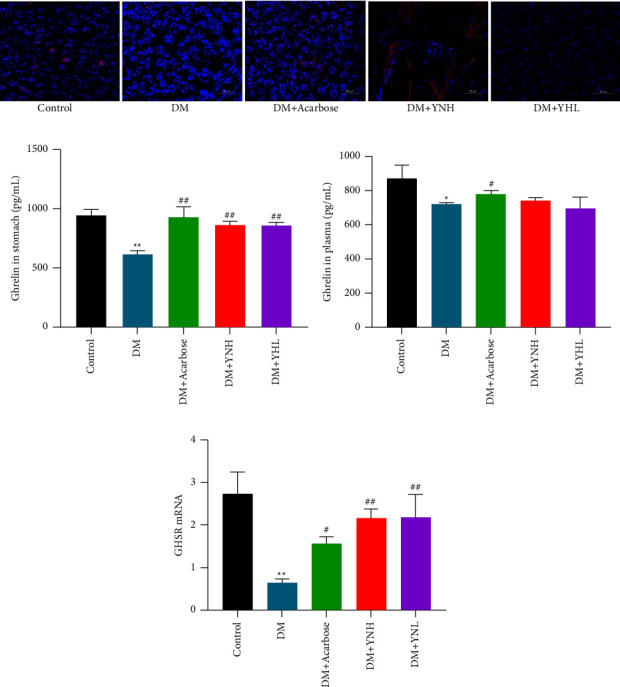
The effect of Yunvjian on ghrelin hormone secretion and expression of GHSR mRNA. (a) Ghrelin immunofluorescence staining of gastric antrum (200×); (b) ghrelin secretion in gastric antrum; (c) ghrelin secretion in serum; (d) GHSR mRNA. The data are expressed as mean ± standard error of mean. The data are expressed as mean ± standard error of mean. Compared with the control group: ^*∗*^*P* < 0.05 and ^*∗∗*^*P* < 0.01; compared with the DM group: ^#^*P* < 0.05 and ^##^*P* < 0.01.

Compared with the control group, the gastric ghrelin level of diabetic rats was significantly reduced (949.3 ± 36.27 vs. 616.2 ± 26.23, *P* < 0.01, Figure 5(b)), but the difference in serum was reduced (873.47 ± 60.85 vs. 722.5 ± 4.75, *P* < 0.05, Figure 5(c)). Compared with the DM group, the stomach and serum ghrelin levels in the DM + Acabose group were significantly increased (616.2 ± 26.23 vs. 926.9 ± 69.85; 722.5 ± 4.75 vs. 784.01 ± 21.74, *P* < 0.01, Figures 5(b) and 5(c)). After administration of Yunvjian, the gastric ghrelin level increased, among which the DM + YNH group increased the most (*P* < 0.01). In order to further examine the influence of Yunvjian on Ghrelin hormone secretion, a fluorescent fluorescence test was performed on the gastric antrum. The red fluorescence of the DM group was weakened, and the red fluorescence of DM + Acabose and DM + YNH increased than the control group, suggesting that the high concentration of Yunvjian can promote the secretion of Ghrelin hormone, while the low concentration has no obvious change (Figure 5(a)).

Figure 5(d) shows the expression of Ghrelin mRNA in the stomach of DM rats decreased significantly compared with the control group (*P* < 0.05). Compared with the DM group, the expression of Ghrelin mRNA in the stomach of the DM + Acarbose group was significantly increased (*P* < 0.01); the expression of Ghrelin mRNA in the stomach of the DM + YNH and DM + YNL groups also increased significantly (*P* < 0.01), and more than the DM + Acarbose group, but the difference was not significant (*P* > 0.05). The effect of Yunvjian up-regulating GRSH mRNA is more obvious.

## 4. Discussion

Diabetes mellitus is a type of systemic metabolic disease characterized by increased glucose caused by genetic, environmental, and other factors [18]. As the course of the disease continues to progress, it is easy to cause a variety of complications [19].

The main pathogenesis of T2DM is due to the malfunction of the kidneys, stomach, and spleen organ systems according to TCM theory. Yunvjian is composed of *Rehmannia glutinosa* Libosch. (Shu Di Huang), *Achyranthes bidentata* Bl. (Niu Xi), Gypsum Fibrosum (Shi Gao), *Anemarrhena asphodeloides* Bge. (Zhi Mu) and *Ophiopogon japonicus* Ker-Gawl. (Mai Dong), which is a classic TCM with the effect of nourishing kidney yin and clearing stomach heat. Pharmacological investigations show, *Rehjnannia glutinosa* Libosch. (Shu Di Huang) contains catalpol and acteoside, possess a bioactivities such as antidiabetes and antiosteoporosis [20]. Research shows that, the extract of the *Achyranthes bidentata* Bl. (Niu Xi) significantly improved FPG, INS, NOXs, ROS, SOD, and other indicators of T2DM rats [21]. *Anemarrhena asphodeloides* Bge. (Zhi Mu) and its main components have shown anti-diabetic effects [22]. The main active ingredient of *Ophiopogon japonicus* Ker-Gawl. (Mai Dong) is ophiopogon polysaccharide, which has been proved to have protective effects on diabetes, cardiovascular, and chronic inflammatory diseases [23]. Therefore, we speculate that Yunvjian has the function of controlling glucose metabolism and relieve symptoms of diabetes.

In this study, DM rats showed impaired glucose tolerance and stable fasting hyperglycemia, which was improved by administration of acarbose and Yunvjian for 4 weeks. As same as some current studies [24], in our experiment, we found that the fasting insulin level of diabetes rats increased, suggested Yunvjian reduced the HOMA-IR in DM rats. The active ingredients of Yunvjian, such as stigmasterol, have certain therapeutic effects on hyperglycemia [25]. In this study, in DM rats, Yunvjian treatment can relieve the impairment of pancreas cells. We think this may be one of the reasons why Yunvjian has the antidiabetes effect.

Gastrointestinal motility disorder is a common complication of diabetes, accounting for about 40% of diabetic patients [11]. Based on the evidence that Yunvjian can regulate blood glucose and protect islet *β* cells, it is further clarified whether intervention of gastric emptying function can have a positive effect on improving insulin resistance, which may bring new inspiration for the prevention and treatment of diabetes and complications. As the pacemaker cell of gastric peristalsis [26], ICC can maintain the contraction of smooth muscle and peristalsis of the stomach, and regulate the voluntary rhythmic movement of the stomach [27]. It plays a role in transmitting signals to the smooth muscle during the emptying process of the stomach [28]. Receptor tyrosine kinase (c-kit) is the key to maintaining the phenotype of ICC, which can specifically observe the distribution and expression of ICC in the gastrointestinal tract [29]. When c-kit is blocked by antibodies, it will be found that ICC cannot develop normally [30]. In this experiment, we use SPECT to detect gastric emptying. This study showed that, half emptying time in DM rats were significantly increased compared with Control group, while 1 h at the end of the study, the gastric emptying rate and the number of cajal was significantly decreased. Compared with DM rats, Yunvjian treatment can recovery the decreased levels of ICC with accelerate gastric emptying rate.

Ghrelin is mainly produced in the gastrointestinal polypeptide [31], and is also distributed in organs such as the hypothalamus, pituitary gland, and islets [32]. It can promote the secretion of growth hormone, promote the emptying of the stomach and the peristalsis of the small intestine [33]. Ghrelin also can promote insulin secretion in mouse pancreatic islet *β* cells, and evidences show that Ghrelin hormone can increase its insulin secretion [34]. GHSR is a G protein-coupled receptor that binds to Ghrelin on the cell surface. Ghrelin hormone and GHSR can only show their effects after being combined with GHSR [35]. Our study found that the levels of Ghrelin and GHSR mRNA in diabetes rats decreased significantly, while after 4 weeks administration, Yunvjian promoted Ghrelin and GHSR mRNA level. In this study, we confirm that Yunvjian has a protective effect on the development of diabetes by involves changes in gastric emptying function.

## 5. Conclusion

Yunvjian treatment can significantly improve glucose metabolism and pancreatic islet function in T2DM rats, and can significantly improve the morphology and function of pancreatic *β*-cells. Its protective effect on pancreatic *β*-cells may lie in promoting gastric emptying function, repairing gastric antrum smooth muscle damage, improving the number and morphology of Cajal cells, and promoting the release of gastric Ghrelin hormone and the expression of GHRS mRNA levels. The results of this study will provide the foundation for further research for the hypoglycemic mechanism and medicinal value of Yunvjian.

## Figures and Tables

**Table 1:** Primer design of GHSR.

Genes	Accession no	Primer sequence	
GAPDH	NM_017008.4	S 5′CTGGAGAAACCTGCCAAGTATG3′	
		A 5′GGTGGAAGAATGGGAGTTGCT3′	

GHSR	NM_032075.3	S 5′GTGTCCAGCGTCTTCTTCTTTC3′	
		A 5′CACCACAGCAAGCATCTTCACT3′	

## Data Availability

The original data presented in the study will be available in the article/Supplementary Material, further inquiries can be directed to the corresponding author.
